# Disentangling the causes of high polymorphism sharing in sympatric *Petunia* species from subtropical highland grasslands: insights from nuclear diversity

**DOI:** 10.1590/1678-4685-GMB-2023-0159

**Published:** 2023-10-30

**Authors:** Luize Simon, Luana S. Soares, Loreta B. Freitas

**Affiliations:** 1 Universidade Federal do Rio Grande do Sul, Departamento de Genética, Porto Alegre, RS, Brazil.

**Keywords:** Petunia, Solanaceae, genetic diversity, speciation, subtropical highland grassland

## Abstract

Genetic polymorphism sharing between closely related and sympatric plant species could result from common ancestry, ancient or recent hybridization. Here we analyzed four *Petunia* species from the subtropical highland grasslands in southern South America based on nuclear diversity to disentangle the causes of high polymorphism sharing between them. We genotyped microsatellite loci, employed population genetic methods to estimate variability, species limits, and ancient and recent gene flow, and assigned individuals to genetic and taxonomic groups. Finally, we modeled evolutionary processes to determine the impact of Quaternary climate changes on species phylogenetic relationships. Our results indicated that genetic diversity was strongly influenced by expansion and habitat fragmentation during the Quaternary cycles. The extensive polymorphism sharing is mainly due to species’ common ancestry, and we did not discard ancient hybridization. Nowadays, niche differentiation is the primary driver for maintaining genetic and morphological limits between the four analysed *Petunia* species and there is no recent gene flow between them.

## Introduction

Closely related and recently diverged plant species usually share many features, such as morphological traits, ecological requirements, and genetic polymorphisms. The degree of divergence between these species depends on the time since separation, the intensity and direction of remaining gene flow, if any, and the strength of stochastic and directed evolutionary processes (e.g., Ley and Hardy, 2014). Thus, polymorphism sharing might be an indication of a shared recent common ancestor, selection pressure, or hybridization (Comes and Abbott, 2001).

Hybridization, defined as the gene flow between different lineages or species (Gompert and Buerkle, 2016), can have contrasting consequences. On the one hand, hybridization can lead to the extinction of the lineages as they are known (e.g., Seehausen, 2013), on the other hand, it may produce genetic combinations able to colonize new environments (e.g., Burgarella et al., 2019). Independently of its effects, hybridization is more probable as the lineages and species are evolutionarily closer (Abbott et al., 2013). Changes in the landscape can be a driver for species range expansion and isolation due to fragmentation throughout their distribution. For example, expansion may put different species or lineages in contact, which favors hybridization under suitable conditions such as weak reproductive barriers and pollinator sharing (Schley et al., 2022).

A recent review (Turchetto et al., 2022) highlighted the role of biotic and abiotic drivers for hybridization in plants from the Neotropics. Pleistocene climate changes were involved in most of the revised examples, whereas some pollinators’ behaviors were pivotal as biotic drivers. The past climate changes during the Pleistocene affected plant distribution (Behling, 2002), diversity, and population structure in the southern hemisphere (e.g., Barros et al., 2015, 2020; Backes et al., 2019; John et al., 2019). The Quaternary climate shifts influenced plant range due to the forest contraction and grassland expansion during the glacial periods and migration in a converse sense at interglacial phases (Carnaval et al., 2009), affecting several plant groups (e.g., Lorenz-Lemke et al., 2010; Barros *et al.*, 2015). The climate cycle alternation produced landscape fragmentation and, in some areas, resulted in vegetation mosaics among grasslands and forests (Behling and Pillar, 2007).

In the subtropical highland grasslands in southern Brazil (SHGs), vegetation is a mosaic between grassland-adapted species and Araucaria Forest, with *Araucaria angustifolia* as the dominant tree species. SHGs are located throughout the Serra Geral, inserted in the Brazilian Atlantic Forest domain, and display high diversity in geological substrates and altitudinal variation (Behling, 2002). SHGs were strongly affected by the Pleistocene climate cycles, which shaped the current landscape and species structure and diversity, allowing rapid speciation for different plant groups (Barros et al., 2015, 2020), mainly in allopatric processes (Lorenz-Lemke et al., 2010; Fregonezi et al., 2013; Mäder and Freitas, 2019). The climate shifts and vegetation contraction-expansion dynamic are often pointed out as the primary driver of species richness and endemism observed in SHGs (Iganci et al., 2011). In turn, isolation can interrupt the gene flow and, if it occurs rapidly and intensely, promotes speciation with low differentiation (Binelli et al., 2020), intensifying the speciation rates (e.g., Lorenz-Lemke *et al.*, 2010; Mäder and Freitas, 2019). 

**Figure 1 -:**
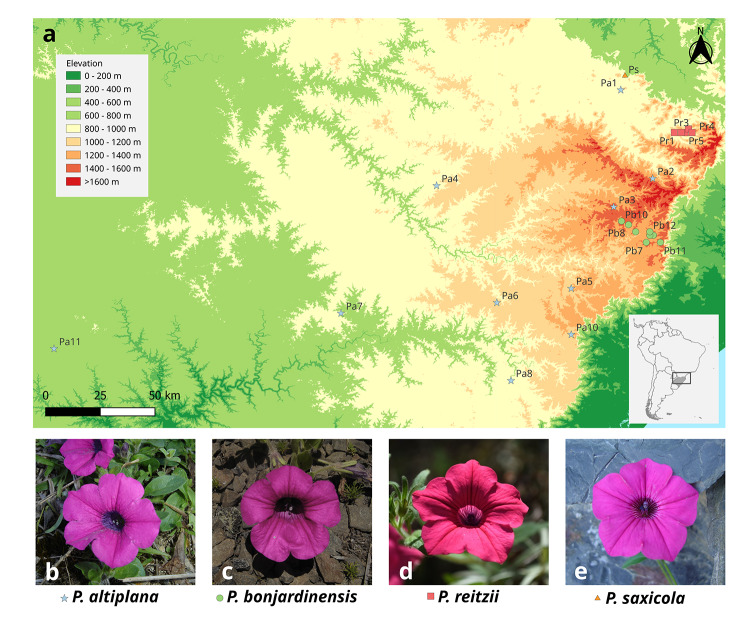
Sampling distribution and analyzed species. (a) All collection sites location: blue stars, *P. altiplana*; green dots, *P. bonjardinensis*; pink squares, *P. reitzii*; orange triangles, *P. saxicola*. (b) to (e) flowers in frontal view of each species (photos by J.R. Stehmann, UFMG).

The genus *Petunia* (Solanaceae) is a young herbaceous group that originated and diversified in lowland grasslands in southern South America, migrating to highland grasslands as Pleistocene climate cycles favored herbs to expand (Reck-Kortmann et al., 2014). Four *Petunia* species are restricted to the SHG (Figure 1): *P. altiplana* has the most extensive distribution, reaching 300-1,500 m above sea level (a.s.l.) in open fields in Rio Grande do Sul and Santa Catarina Brazilian states, can be found in open and sunny grasslands; *P. bonjardinensis* that grows in open and disturbed areas, at 1,200-1,500 m a.s.l. in Santa Catarina; *P. reitzii* that occurs at 800-1000 m a.s.l. growing on the walls of small cliffs beside rivers, hanging freely in space in Santa Catarina; and *P. saxicola,* which inhabits humid and shadowed rocks, in the forest borders, at 800 m in elevation also only in Santa Catarina (Stehmann et al., 2009; Lorenz-Lemke et al., 2010; Souza et al., 2022; Soares et al., 2023).

Although the four species have a sympatric distribution considering most of their occurrence area, they are never found in the same site, occupying different micro-environments. These species are interfertile, as demonstrated by experimental pollinations and bee-pollinated, with the same solitary bees’ genera already observed visiting their flowers (Stehmann et al., 2009). Nuclear and plastid data were previously published for these species revealing high polymorphism sharing among them (Lorenz-Lemke et al., 2010; Souza et al., 2022; Soares et al., 2023). Species share a common ancestor (Reck-Kortmann et al., 2014), diverged recently, and evolved under the Pleistocene influence in an allopatric process due to landscape fragmentation (Lorenz-Lemke *et al.*, 2010). Moreover, has been suggested that the nuclear polymorphism sharing could be due to hybridization with another *Petunia* species widely distributed in the same area (Souza *et al.*, 2022). Testing such hypothesis regarding multiple hybridization events under landscape changes served as starting point to re-evaluate the microsatellite data.

The landscape fragmentation and range expansion, mainly those resulting from climate changes in SHG, could have put the four *Petunia* species in contact facilitating hybridization, as there is incomplete reproductive isolation among *Petunia* species (Watanabe et al., 2001). Such a scenario could explain the polymorphism sharing due to hybridization (Payseur and Rieseberg, 2016). Instead, the same landscape changes could allow divergence under a sky-island model (Oliveira et al., 2021) for species that were kept isolated during the expansion and contraction cycles.

Here, we aimed to answer the following questions: (1) Did hybridization occur among these *Petunia* species explaining the polymorphism sharing? (2) Was hybridization an ancient or recent event? (3) If hybridization does not explain the polymorphism sharing, which alternative evolutionary driver does?

## Material and Methods

### Plant material and genetic information

In this work, we included 38 individuals of *P. altiplana*, 30 of *P. bonjardinensis*, 20 of *P. reitzii*, and 22 of *P. saxicola* (Table S1). Sampling size was proportional to the species density and range. We retrieved genetic information based on eight nuclear microsatellites, amplified with previously published primers (Bossolini et al., 2011; Table S2). We re-evaluated the original data (Souza et al., 2022; Soares et al., 2023) by visualizing and scoring the alleles with GeneMarker v.1.97 software (Softgenetics LLC, State College, USA). We used Micro-Checker (van Oosterhout et al., 2004) to identify possible null alleles, significant allele dropout, and scoring errors due to stutter peaks. 

We evaluated basic genetic diversity indices such as the number of alleles per locus (A), allele richness (AR), gene diversity (GD), and inbreeding coefﬁcient (*F*_*IS*_ ) per locus using Fstat v.2.9.4 (Goudet, 1995). The levels of observed (*H*_*O*_ ), expected (*H*_*E*_ ), and total (*H*_*T*_ ) heterozygosity, and any signiﬁcant deviations from the Hardy-Weinberg equilibrium (HWE) after Bonferroni correction were estimated in Arlequin v.3.5.2.2 (Excoffier and Lischer, 2010). Finally, we computed the genetic differentiation using a hierarchical analysis of molecular variance (AMOVA; Excoffier *et al.*, 1992) in Arlequin to estimate genetic variation between taxa.

### Individual identity and gene flow

We estimated the individual identity based on genetic structure among species using the discriminant analysis of principal components (DAPC; Jombart et al., 2010) performed in Adegenet (Jombart, 2008). We performed the analysis without including any geographical location or taxonomic priors and used the *find.clusters* option to identify the clusters under a Bayesian information criterion (BIC) and the function *optim.a.score* to find the best number of principal components to keep. The group membership probabilities were visualized using the *compoplot* function.

We also ran Structure v.2.3 (Pritchard et al., 2000) with no previous information about each sample’s taxonomy or geographical origin. We estimated the best *K* value through ∆*K* (Evanno et al., 2005) in Harvester v.0.6.93 (Earl and von Holdt, 2012). We performed Structure using 10^6^ Markov chain Monte Carlo (MCMC) repetitions after a 2.5 x 10^5^ burn-in period and ten iterations per *K* value, evaluating different numbers of clusters (1 to 5). The resulting bar plot from the summarized iterations for the best *K* was generated using Pophelper (Francis, 2017).

We used NewHybrids v.1.1 (Anderson and Thompson, 2002) software to estimate the probability of individuals belonging to distinct hybrid or purebred classes (F1, F2, backcrosses with each considered parental species). We ran two independent analyses using Jeffrey’s priors with uniform priors that included 10^5^ steps as burn-in followed by 10^6^ MCMC interactions to ensure the chains’ convergence and homogeneity across runs.

We performed historical and contemporary estimates of gene flow between the species using the coalescent-based method implemented in Migrate-N v.3.6.11 (Beerli and Felsenstein, 2001; Beerli, 2006) and the disequilibrium-based method implemented in BayesAss v.3.0.4 (Wilson and Rannala, 2003). In Migrate-N, we estimated ancient migration (m) and effective population size (*N*e) between the species using the Brownian motion model, with starting conditions based on 10% of the prior and uniform prior distribution to estimate theta (θ) and M, which is the migration rate in θ scale. We included one long chain, with 1,000 sampling steps, 200,000 recorded genealogies, and 500 chains as burn-in. In addition, we ran four independent chains with different temperatures (1.0, 1.5, 3.0, and 1 x 10^6^) in a Metropolis-coupled MCMC (MCMCMC) procedure (Geyer and Thompson, 1995) to ensure better mixing along the chains. We calculated the estimates per locus and summarized them as weighted values for all loci. Finally, we assessed the MCMC stationarity by checking the posterior distribution over all loci and running the analysis several times with different starting points. To obtain Ne, we used θ values with SSR mean mutation rate (μ) uniformly ranging from 10^-4^ to 10^-2^ (Zhang and Hewitt, 2003); to estimate the number of migrants, we used M with recipient population θ value [(Ne = θ / 4μ) and (m = M x θ)].

In BayesAss, we estimated current gene flow by performing the analysis in triplicate using different seeds. We ran each round for 1 x 10^7^ iterations, sampling every 100 iterations, with a burn-in of 1 x 10^6^ iterations. To achieve acceptance rates between 20 and 40 %, we set the mixing parameters for migration rate (0.2), inbreeding (0.5), and allele frequency (0.5). Finally, we assessed the convergence of the MCMC using Tracer v.1.7.1 (Rambaut et al., 2018) and chose the lowest Bayesian deviance value per run (Faubet et al., 2007; Meirmans, 2014).

**Figure 2 -:**
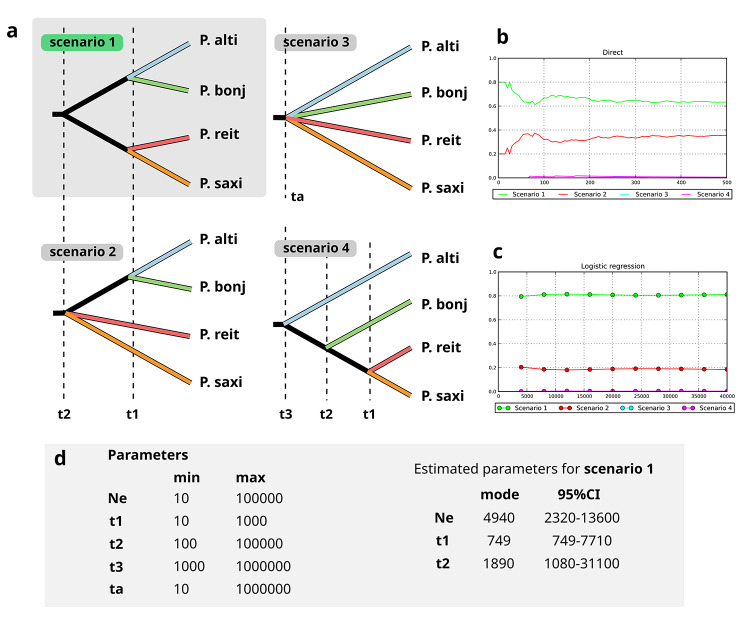
Evolutionary modeling analysis using ABC approach in DYABC software for four *Petunia* species from Subtropical Highland Grasslands. (a) four tested scenarios, highlighting the best-model (scenario 1); (b) scenarios’ posterior probability assessed based on logistic regression; (c) scenarios’ posterior probability assessed based on logistic approach; (d) parameters’ priors for all scenarios and estimated parameters for the best-model (scenario 1). Scenarios 3 and 4 had zero posterior probability in both approaches.

To understand the evolutionary history of these four *Petunia* species, inferring the divergence time and ancestral population effective size, we analyzed four alternative scenarios (Figure 2a) for taxa divergence using an approximate Bayesian computation (ABC) approach in DIYABC v.2.0 (Cornuet et al., 2014). The scenarios changed in how the species diverged and the different phylogenetic relationships between species. The divergence time in all scenarios included the last glacial maximum, when grasslands dominated the SHG, indicating a cold and dry climate. These scenarios also differed in the number and order of divergence events, and we did not consider any change in effective population size. Scenario 1 involved the ancestral population diverging into two subpopulations, one giving rise to *P. altiplana* and *P. bonjardinensis* and another to *P. reitzii* and *P. saxicola* group. In scenario 2, the ancestral population diverged into three subpopulations, one corresponding to *P. reitzii,* the second to *P. saxicola* independently, and the last grouped *P. altiplana* and *P. bonjardinensis*. In scenario 3, the ancestral population diverged independently into four lineages, one for each species. Scenario 4 involved a successional divergence, first *P. altiplana,* second *P. bonjardinensis,* and, finally, *P. reitzii* and *P. saxicola* group. We estimated the divergence time for each scenario using priors uniformly distributed between the minimum and maximum values (Figure 2D). We assumed generation time as one year, as *Petunia* species are annual herbs (Stehmann et al., 2009).

We simulated one million data sets for each scenario. We used 30 summary statistics to represent the observed data per species separately (mean number of alleles, gene diversity, allele size, and size variance) and for species pairs (mean number of alleles, gene diversity, and *F*_*ST*_ )_._ Each scenario’s posterior probability (PP) was assessed based on logistic regression and direct estimate approaches using the 10,000 and 100 best simulations. For the best scenario, the parameters’ posterior distribution was estimated using the *logit* transformation and considering the 10,000 best simulations. We used several strategies to assess the confidence in scenario choice and parameter estimation. First, we perform a posterior model checking based on 10,000 simulations. The Type I error was estimated as the proportion of incorrect exclusion of the true model among the datasets simulated under a given scenario. The Type II error was calculated as the recovery rate of a given scenario when it was not the true model (Cornuet et al., 2010). We also determined the bias and precision of parameter estimation based on 1,000 pseudo-observed datasets with parameters sampled from the posterior distribution.

## Results

### Genetic diversity

All loci exhibited a clear single band per allele. All individuals displayed one or two alleles per locus, consistent with the diploid condition of *Petunia* species and with the expected sizes based on loci description. Null alleles were present in low frequency (< 0.5%), and no significant allele dropout and scoring errors due to stutter peaks were observed considering the eight analyzed loci. All pairs of loci were in linkage equilibrium after Bonferroni’s correction (P < 0.001), and levels of polymorphism and diversity were high for most of the eight microsatellite loci (Table S3). The combined probability of identity (PID) for the eight loci was almost zero for all taxa, indicating that two unrelated individuals would not share the same multilocus genotype.

Comparing the four species, *P. altiplana* showed the highest diversity values considering allele richness, and gene diversity, whereas *P. saxicola* had the lowest indices. Observed heterozygosity was lower than expected under Hardy-Weinberg equilibrium (P < 0.05), except for *P. saxicola.* The highest heterozygotes deficit was observed in *P. altiplana*. All species had significant *F*_*IS*_ values, negative only for *P. saxicola*. The four species displayed private alleles that were more frequent in *P. altiplana* (Table 1). Comparing the four species based on genetic structure, AMOVA showed that most variation is observed within populations (83%), whereas *F*_*ST*_ between species did not reveal differences between *P. bonjardinensis* and *P. reitzii* (*F*_*ST*_ = 0.06; P < 0.01).

**Table 1 -:** Genetic diversity median values for four *Petunia* species based on nuclear microsatellites.

Species	N	A	AR	P	GD	H_E_	H_O_	F_*IS*_
*P. altiplana*	38	76	7.03	21	0.66	0.66	**0.48**	**0.27**
*P. bonjardinensis*	30	60	6.16	12	0.58	0.61	**0.51**	**0.14**
*P. reitzii*	20	40	4.62	4	0.50	0.58	**0.50**	**0.16**
*P. saxicola*	22	46	4.06	4	0.47	0.53	**0.61**	**-0.26**

N - number of analyzed individuals; A - total number of alleles per species; P - number of private alleles; AR - allele richness (min. sample size of 14 individuals); GD - gene diversity per locus; H_E_ - expected heterozygosity; H_O_ - observed heterozygosity; *F*_*IS*_ - inbreeding coefficient. Bold values indicate significant HWE deviation (P < 0.05) after Bonferroni’s correction.

### Individual identity and gene flow

**Figure 3 -:**
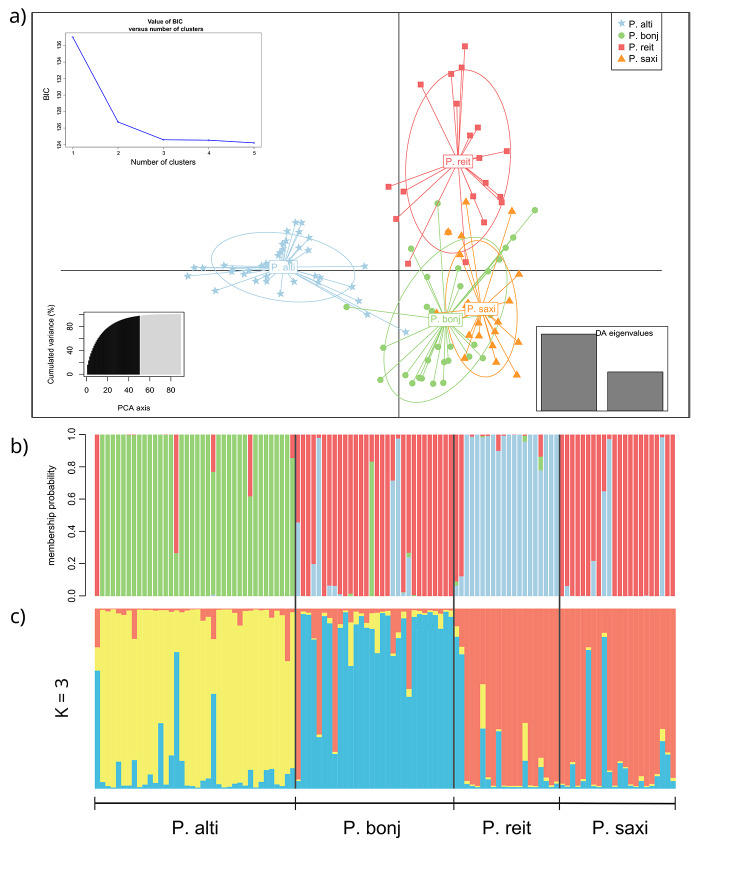
Clustering analyses for individuals of four *Petunia* species based on microsatellite variability. (a) DAPC Cartesian plane with individuals colored according their taxonomic classification as lables at the top; (b) DAPC scatter plot indicating the membership probability; (c) Bar plot considering the best number of genetic components obtained with Structure software (K = 3). In (b) and (c), each vertical bar represents individuals and colors indicate the genetic components; solid black bars separate species; individuals with q ≥ 0.2 were considered mixed.

The DAPC analysis (Figure 3A) identified three independent groups. Individuals classified as *P. altiplana* or *P. reitzii* formed separate clusters, whereas *P. bonjardinensis* and *P. saxicola* formed the third group. We retained seven principal components (PCs) and three discriminant eigenvalues, resulting in three genetic components with more than one (q ≥ 0.2) per species (Figure 3B). Structure analysis also revealed three genetic components (best K = 3). Still, contrarily to DAPC, one component predominated among *P. altiplana* individuals, another was associated with *P. bonjardinensis*, and the third was shared by *P. reitzii* and *P. saxicola*. Several individuals displayed mixed genetic constitutions, and none had only the genetic component of different species (Figure 3C). All individuals displaying improper membership probability in DAPC presented q ≥ 0.2 in Structure, although several other individuals have been assigned as admixed. All admixed individuals showed the *P. bonjardinensis* genetic component. Just a few individuals display polymorphism of the three clusters.

The NewHybrids analysis (Figure S1) revealed that no individual, even those with admixed ancestry in Structure, was classified as F_1_ hybrid and, overall, most were purebred. We find individuals classified as *P. saxicola* purebred among *P. bonjardinensis* (one) or *P. reitzii* (three) individuals.

The Migrate-N estimation for the ancestral population size (Table 2) varied between species. The highest Ne value was observed for *P. saxicola* and the lowest for *P. bonjardinensis*. We observed ancient bidirectional gene flow among all species. In contrast, BayesAss revealed most of the individuals were residents of each species (M ≥ 0.93 ± 0.03), and a low or no recent gene flow was observed between species (Table 2).

**Table 2 -:** Effective population size and migration among the highland species estimated by Migrate-N and BayesAss.

		M				Θ	Ne 10-4	Ne 10-2
		*P. alti*	*P. bonj*	*P. reit*	*P. saxi*			
**Migrate-n**	**P. alti**		100.33	99.00	98.33	1.30	3250.00	32.50
	**P. bonj**	96.33		91.67	100.33	1.10	2750.00	27.50
	**P. reit**	94.33	98.33		103.00	1.37	3416.68	34.17
	**P. saxi**	96.33	101.67	100.33		1.50	3750.00	37.50
**BayesAss**	**P. alti**	**0.97(0.02)**	0.01(0.01)	0.01(0.01)	0.01(0.01)			
	**P. bonj**	0.01(0.01)	**0.95(0.03)**	0.02(0.02)	0.02(0.02)			
	**P. reit**	0.02(0.02)	0.03(0.02)	**0.93(0.03)**	0.03(0.02)			
	**P. saxi**	0.01(0.01)	0.02(0.02)	0.02(0.01)	**0.95(0.02)**			

M - Migration rate; θ - theta; Ne - effective size of founder population. Bold values indicate intraspecific gene flow in BayesAss analysis; values in parenthesis correspond to the standard deviation. Cells in blue, migration from - to; in orange, opposite direction

Evolutionary modeling through ABC analysis (Figure 2A) revealed that the best model was scenario 1 (Figure 2B, C), which stated the divergence of two sister lineages that gave rise to a group with *P. altiplana* and *P. bonjardinensis* and another clustering *P. reitzii* and *P. saxicola*. The four species have diverged simultaneously during t1 (included in the Quaternary period).

## Discussion

Here we investigated the potential drivers for polymorphism sharing in four *Petunia* species from the subtropical highland grasslands in southern Brazil, basing our population diversity and structure analyses on nuclear microsatellites. First, we hypothesized that the polymorphism sharing among the four species was due to interspecific hybridization, as hybridization is more likely between closely related species (Whitney et al., 2010) for which barriers against gene flow are incomplete or absent, and species are in contact or nearly distributed (Schley et al., 2022). Alternatively, we considered a recent common ancestry to explain the high genetic similarity between species. Finally, we did not consider common selective forces because at least three species have nonoverlapping ecological niches (Souza et al., 2022).

The four SHG *Petunia* species displayed genetic diversity (Table 1) compatible with their geographical range compared with other *Petunia* species and similar markers (e.g., Turchetto et al., 2015b; Silva-Arias et al., 2017; Backes et al., 2019) and following the general statement for correlation between the extent of distribution and diversity indices (Gitzendanner and Soltis, 2000). The most diverse species was *P. altiplana,* which has the largest distribution area (Soares et al., 2023). The lowest variability values were observed for *P. saxicola,* found in only one location (Souza et al., 2022).

Three in four species showed homozygote excess, indicating significant inbreeding values probably due to biparental inbreeding because these species are considered self-incompatible (Robertson et al., 2011), and most *Petunia* species have pollen dispersal at short distances (e.g., Turchetto et al., 2015a). Moreover, populations of these three species are distantly distributed, and just a few individuals compose them (Souza et al., 2022; Soares et al., 2023). Only *P. saxicola* showed an excess of heterozygotes that could be attributed to the self-incompatibility in this species (Robertson *et al.*, 2011) and the massive presence of individuals as the species currently occurs in only a known site (Souza *et al.*, 2022).

The four SHG *Petunia* species are not syntopic (Lorenz-Lemke et al., 2010; Souza et al., 2022; Soares et al., 2023), each occurring in different microenvironments. All these species are bee-pollinated, and the same generalist bee genera have been observed visiting them (Stehmann et al., 2009). Such bees have flight ranges compatible with distances between species (Mouga et al., 2016), which makes the interspecific gene flow between *Petunia* species from SHG theoretically likely. However, current mating events do not seem to contribute to the polymorphism sharing between species, which, according to our results, has been restricted to the past. The membership probability in DAPC analysis indicated that a few individuals were assigned to different species than expected according to their taxonomic classification or provenience (Figure 3B). Most individuals showed mixed ancestry in Structure analysis (Figure 3C), especially involving the *P. bonjardinensis* genetic component. Based on the plastid haplotypes (Lorenz-Lemke *et al.*, 2010), *P. bonjardinensis* shares haplotypes only with *P. altiplana*, whereas *P. altiplana* shares with the other three, and *P. reitzii* and *P. saxicola* share all their haplotypes. In the molecular phylogenetic tree (Reck-Kortmann et al., 2014), *P. reitzii* and *P. saxicola* have the most recent common ancestor, whereas *P. bonjardinensis* is basal to them and *P. altiplana* that belongs to the same subclade has a species from tropical grassland as the sister group, occupying a more basal position to the other three SHG species. The strong phylogenetic signal was confirmed here mainly regarding *P. reitzii* and *P. saxicola*, which shared the same genetic component (Figure 2A and Figure 3C) and occupied the same genospace in DAPC Cartesian plane with several superimposed individuals (Figure 3A).

The *Petunia* ancestor originated in lowland grasslands (Reck-Kortmann et al., 2014) ca. 3.0 million years ago (Mya; Särkine et al., 2013). Based on the coalescence of plastid haplotypes, the SHG lineage split from sister group ca. 1.3 Mya, still in low to middle elevation grasslands, diversifying during the Quaternary in highland fields ca. 1.0 Mya (Lorenz-Lemke et al., 2010) under a sky-island model that would explain SHG *Petunia* endemism, range limits, and species richness. Our results supported such an evolutionary model where the ancestor lineage expanded its range during cold and dry periods and contracted it in warmer and wetter cycles (Figure 2). Such demographic processes could promote recurrent ancient connections and fragmentation between SHG precursor lineages or even themselves at primary diversification stages allowing hybridization that, summed to the common origin, would explain the polymorphism sharing without current hybridization (Table 2).

The divergence from a common ancestor expanding its range is primarily based on stochastic processes such as drift, mutation, and recombination. Ancestral gene flow detected between *Petunia* species from the SHG is consistent with phylogenetic radiation without strict reproductive isolation, a property of the mechanisms of maintenance of genetic integrity in species complexes as observed in other Neotropical species (e.g., Binelli et al., 2020). Four endemic *Calibrachoa* species also occur in SHG. The genetic variability and population structure of these *Calibrachoa* species (John et al., 2019) revealed a similar differentiation process. Moreover, Quaternary cycles are the main driver for phylogenetic niche conservatism in *Calibrachoa* (Mäder and Freitas, 2019), including the SHG clade.

In conclusion, our results supported the idea that polymorphism sharing between the four SHG *Petunia* species is mainly due to a common ancestral as different clustering analyses combined individuals differently (Figure 3), and only ancestral migration events were detected (Table 2; Figure S1). Despite that, we cannot discard an ancient gene flow between ancestral lineages of these four species. Therefore, a more detailed genomic evaluation should be implemented to disentangle such pending questions.

## Supplementary material

The following online material is available for this article:

Table S1 - Sampling information for four *Petunia* species from the Subtropical Highland Grasslands.

Table S2 - Nuclear microsatellite markers used to genotype four *Petunia* species.

Table S3 - Genetic diversity per locus of nuclear microsatellite considering four *Petunia* species.

Figure S1 - Posterior probabilities of Newhybrids of four *Petunia* species individuals (pure parental species, F1 or F2 hybrids, and backcrosses); classes follow label colors. Vertical dotted black lines separate individuals according their taxonomic classification in each comparison.
