# Supplementary material for: Disentangling the causes of high polymorphism sharing in sympatric Petunia species from subtropical highland grasslands: insights from nuclear diversity
Source: Genet Mol Biol. 2023 Oct 30;46(3 Suppl 1):e20230159. doi: 10.1590/1678-4685-GMB-2023-0159 (PMC10619130; doi:10.1590/1678-4685-GMB-2023-0159)
Supplement: Table S1 - [file 1415-4757-GMB-46-3-s1-e20230159-suppl1.pdf]

Genetics and Molecular Biology
https://doi.org/10.1590/1678-4685-GMB-2023-0159

Supplementary material to "Disentangling the causes of high polymorphism sharing in sympatric *Petunia* species from subtropical highland grasslands: insights from nuclear diversity"

Table S1 - Sampling information for four *Petunia* species from the Subtropical Highland Grasslands.

Species	ID	N	Geographical coordinates	Voucher
P. altiplana	PA1	4	27°39'S/49°45'W	BHCB 99674
	PA2	5	28°04'S/49°36'W	BHCB 96683
	PA3	3	28°12'S/49°47'W	BHCB 99752
	PA4	4	28°06'S/50°37'W	NA
	PA5	3	28°35'S/49°59'W	BHCB104859
	PA6	3	28°39'S/50°20'W	BHCB195625
	PA7	3	28°42'S/51°04'W	NA
	PA8	3	29°01'S/50°16'W	BHCB116998
	PA9	3	29°27'S/50°36'W	BHCB 79906
	PA10	4	28°48'S/49°59'W	BHCB195623
	PA11	3	28°52'S/52°25'W	BHCB114597
P. bonjardinensis	PB1	1	28°19'S/49°41'W	BHCB 80080
	PB2	3	28°20'S/49°37'W	BHCB 80082
	PB3	2	28°20'S/49°36'W	BHCB 80085
	PB4	6	28°20'S/49°36'W	BHCB 80086
	PB5	2	28°20'S/49°37'W	BHCB 80092
	PB6	2	28°22'S/49°38'W	BHCB 80093
	PB7	3	28°17'S/49°43'W	BHCB 80097
	PB8	3	28°16'S/49°45'W	BHCB 80098
	PB9	2	28°16'S/49°45'W	BHCB 99755
	PB10	2	28°22'S/49°34'W	BHCB 99773
	PB11	3	28°19'S/49°37'W	BHCB 99777
	PB12	1	28°19'S/49°37'W	BHCB 99778
P. reitzii	PR1	2	27°51'S/49°29'W	BHCB 80068
	PR2	3	27°51'S/49°26'W	BHCB 80069
	PR3	6	27°50'S/49°25'W	BHCB 80071
	PR4	4	27°51'S/49°24'W	BHCB 80073
	PR5	5	27°51'S/49°27'W	BHCB 80074
P. saxicola	PS	22	27°35'S/49°44'W	BHCB 80065

ID - population code; N - number of individuals per collection site; BHCB - Universidade Federal de Minas Gerais herbarium; NA - not available
