# Supplementary material for: Disentangling the causes of high polymorphism sharing in sympatric Petunia species from subtropical highland grasslands: insights from nuclear diversity
Source: Genet Mol Biol. 2023 Oct 30;46(3 Suppl 1):e20230159. doi: 10.1590/1678-4685-GMB-2023-0159 (PMC10619130; doi:10.1590/1678-4685-GMB-2023-0159)
Supplement: Table S2 - [file 1415-4757-GMB-46-3-s1-e20230159-suppl2.pdf]

Genetics and Molecular Biology
https://doi.org/10.1590/1678-4685-GMB-2023-0159

Supplementary material to "Disentangling the causes of high polymorphism sharing in sympatric *Petunia* species from subtropical highland grasslands: insights from nuclear diversity"

Table S2 - Nuclear microsatellite markers used to genotype four *Petunia* species.

Chr	Locus	Label	*Forward*	*Reverse*
2	PM21	NED	CTACCGGTAGGCAGTAGTTGC	CCTCGACCTTCTTCCTGAC
3	PM191	PET	GGAGAAGATTGTTGGTAAC	GGGAAACGATCTCTTGCTG
4	PM173	FAM	CAGCGCTATCAACAGCAG	GTGAGAGGCAAGTGATTGG
4	PM8	FAM	TCTGCAAACTTCAAAGCCAA	ACATGCCATGCACTTTTGAG
5	PM110	NED	GGTACAGGGCTAGCAGG	CTAGTTGGGTGTTCACAG
5	PM177	NED	CCCTTACTCTCTTCTTCACC	GAACTATGAACCATAGCTCTC
6	PM117	VIC	CCATACCCCATCTTCCACTGG	GGTGGCAACCTTGAGCTCC
7	PM157	PET	GTAGTAGTAGTAACCCCACC	CATCAGAAGCTTCTGGAG

Chr – *Petunia hybrida* chromosome. All forward primers contained the M-13 tail **CACGACGTTGTAAAACGAC** sequence. Primers were described in Bossolini *et al.* (2011), and we followed amplification and genotyping protocols according Souza *et al.* (2022) and Soares *et al.* (2023).
