# Supplementary material for: Disentangling the causes of high polymorphism sharing in sympatric Petunia species from subtropical highland grasslands: insights from nuclear diversity
Source: Genet Mol Biol. 2023 Oct 30;46(3 Suppl 1):e20230159. doi: 10.1590/1678-4685-GMB-2023-0159 (PMC10619130; doi:10.1590/1678-4685-GMB-2023-0159)
Supplement: Table S3 - [file 1415-4757-GMB-46-3-s1-e20230159-suppl3.pdf]

Genetics and Molecular Biology
https://doi.org/10.1590/1678-4685-GMB-2023-0159

Supplementary Material to "Disentangling the causes of high polymorphism sharing in sympatric *Petunia* species from subtropical highland grasslands: insights from nuclear diversity"

Table S3 - Genetic diversity per locus of nuclear microsatellite considering four *Petunia* species.

Locus	A	AR	GD	H_O	H_E	H_T	F_{ST}	F_{IS}
PM08	13	3.50	0.22	0.15	0.23	0.25	0.09	0.34
PM21	16	4.69	0.37	0.39	0.37	0.62	0.40	-0.03
PM110	16	4.25	0.59	0.37	0.59	0.59	0.01	0.37
PM117	35	8.92	0.81	0.75	0.81	0.87	0.07	0.08
PM157	34	9.00	0.81	0.70	0.81	0.87	0.06	0.14
PM173	21	5.50	0.52	0.61	0.53	0.54	0.03	-0.16
PM177	57	15.4	0.86	0.64	0.86	0.92	0.07	0.25
PM191	20	6.55	0.61	0.45	0.61	0.78	0.22	0.27

A – total number of alleles per locus; AR – allele richness; GD – gene diversity per locus; H_O – observed heterozygosity; H_E – expected heterozygosity; H_T – total heterozygosity; F_{ST} – fixation index; F_{IS} – inbreeding coefficient
