# Supplementary material for: Disentangling the causes of high polymorphism sharing in sympatric Petunia species from subtropical highland grasslands: insights from nuclear diversity
Source: Genet Mol Biol. 2023 Oct 30;46(3 Suppl 1):e20230159. doi: 10.1590/1678-4685-GMB-2023-0159 (PMC10619130; doi:10.1590/1678-4685-GMB-2023-0159)
Supplement: Figure S1 - [file 1415-4757-GMB-46-3-s1-e20230159-suppl4.pdf]

Genetics and Molecular Biology
https://doi.org/10.1590/1678-4685-GMB-2023-0159

Supplementary Material to "Disentangling the causes of high polymorphism sharing in sympatric *Petunia* species from subtropical highland grasslands: insights from nuclear diversity"

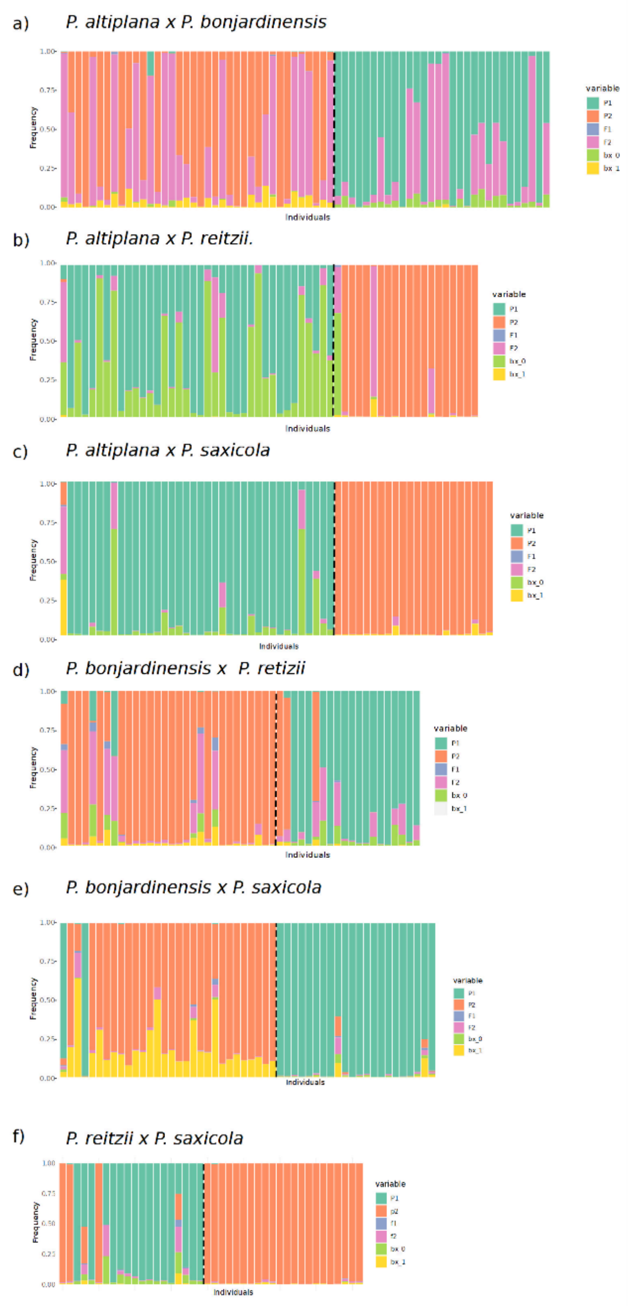

Figure S1 - Posterior probabilities of NEWHYBRIDS of four *Petunia* species individuals (pure parental species, F1 or F2 hybrids, and backcrosses); classes follow label colors. Vertical dotted black lines separate individuals according their taxonomic classification in each comparison.
